# Metformin-associated lactic acidosis and factors associated with 30-day mortality

**DOI:** 10.1371/journal.pone.0273678

**Published:** 2022-08-30

**Authors:** Kanin Thammavaranucupt, Boonchan Phonyangnok, Watanyu Parapiboon, Laddaporn Wongluechai, Watthikorn Pichitporn, Jirut Sumrittivanicha, Somnuek Sungkanuparph, Arkom Nongnuch, Kulapong Jayanama

**Affiliations:** 1 Faculty of Medicine Ramathibodi Hospital, Chakri Naruebodindra Medical Institute, Mahidol University, Samut Prakan, Thailand; 2 Department of Medicine, Nan Hospital, Nan, Thailand; 3 Department of Medicine, Maharat Nakhon Ratchasima Hospital, Nakhon Ratchasima, Thailand; 4 Faculty of Medicine Ramathibodi Hospital, Department of Medicine, Mahidol University, Bangkok, Thailand; The University of the West Indies, JAMAICA

## Abstract

**Background:**

Metformin-associated lactic acidosis (MALA) is a rare event but underrecognition may lead to unfavorable outcomes in type 2 diabetes patients. While many risk factors of MALA have been identified, how to reduce mortality from MALA is a matter of debate. This study aimed to explore the factors associated with 30-day mortality amongst MALA patients.

**Methods:**

An observational study enrolled patients diagnosed with MALA between January 2014 and December 2017. MALA was defined by a history of metformin administration, metabolic acidosis (arterial blood gas pH <7.35 or HCO_3_ <15 mmol/L), and elevated plasma lactate level (>5 mmol/L). We examined risk factors including age, sex, underlying diseases, current medications, blood tests, disease severity, and dialysis data. Mortality status was identified from medical records or report on telephone.

**Results:**

We included 105 MALA patients. Most patients (95.2%) were diagnosed acute kidney injury stage 3 according to KDIGO 2012 definition. The 30-day mortality rate was 36.2% and dialysis rate was 85.7%. The survivors had higher proportions of underlying chronic kidney disease, presence of metabolic acidosis, receiving renal replacement therapy within 6 hours, and haemodialysis, whereas the non-survivors had higher percentage of hypertension and disease severity. Lower APACHE II score (HR = 0.95; 95%CI, 0.91–0.99; p = 0.038), time to dialysis < 6 hours (0.31; 0.14–0.69; 0.004), and haemodialysis (0.20;0.06–0.67; 0.010) were associated with lower 30-day mortality, using multivariate Cox-regression analysis.

**Conclusions:**

Mortality rate amongst patients with MALA was high. Early dialysis treatment within 6 hours after admission and haemodialysis were independently associated with lower 30-day mortality. The large scale, well-designed studies need to confirm these encouraging results.

## Background

Metformin, an anti-hyperglycemic agent of biguanide family, is recommended as a first-line pharmacologic treatment for type 2 diabetes owing to effective glycemic control at reasonable cost [1,2]. Even if metformin is generally prescribed and considered to be safe [3], this anti-hyperglycemic agent can cause life-threatening conditions, especially metformin-associated lactic acidosis (MALA).

MALA is an adverse drug event that presents with high anion gap metabolic acidosis and elevated serum lactate levels without signs of hypoperfusion, in combination with history of metformin administration [4]. Regardless of low incidence (2–47 cases per 100,000 people per year) [5,6], the mortality rate of MALA was considerable, as reported up to 30–50% [7,8]. Previous studies revealed severe dehydration, renal or liver impairment, and sepsis were risk factors for MALA [9–11]. While consensus on treatment of MALA has yet to be determined, metformin cessation and supportive care including normalizing the acid-base balance, discontinuing nephrotoxic agents, and treatment of concomitant diseases were generally done in practice [10]. In order to remove metformin and lactate from blood circulation, kidney replacement therapies (KRT) were taken into account of MALA treatment, especially for patients with severe metabolic acidosis [7,10]. Since prolonged lactic acidosis resulted in organ failure and disease mortality [12], early KRT could result in favorable outcomes. Concerning mode of dialysis, haemodialysis (HD) could be more advantageous due to greater effectiveness in metformin and lactate clearance, compared to peritoneal dialysis (PD) [10].

To date, few studies showed high serum lactate levels, high acute physiology and chronic health evaluation (APACHE) II score, presence of shock, and requirement for mechanical ventilation and vasopressors were associated with increased mortality rate [13–15]. However, understanding of fatal risk factors in patients with MALA (e.g., mode of dialysis, time to dialysis) was limited. Evidence in potential risk factors can improve MALA care plan and decrease MALA associated mortality. This study aimed to explore the factors associated with 30-day mortality amongst MALA patients.

## Materials and methods

### Patients and study design

An observational study enrolled 105 patients diagnosed with MALA and admitted in Maharat Nakhon Ratchasima Hospital, Nakhon Ratchasima, Thailand between January 2014 and December 2017. All patients were 18 years or older. Baseline demographic data including underlying diseases, current medications and personal history were recorded and severity of disease using APACHE II score [16] were evaluated on admission. Blood tests including serum blood urea nitrogen (BUN), creatinine, electrolyte, lactate, and arterial blood gas were collected on admission. Serum BUN and creatinine levels were also collected during hospitalization. Mortality status was identified from medical records or report on telephone until 30 days after admission, following the hospital MALA protocol. Survival time was counted from the date of MALA diagnosis to the death event or 30 days after admission. The study protocol was reviewed and approved by the institutional review boards (IRB), Maharat Nakhon Ratchasima Hospital, Nakhon Ratchasima, Thailand (No. 114/2021). We accessed and analyzed data after receiving the IRB approval (November 2021). All methods were performed in accordance with the relevant guidelines and regulations. Due to the retrospective nature of the study, informed consent was waived.

### Diagnostic criteria

MALA was defined by having all of the following criteria: 1) a history of metformin administration, 2) metabolic acidosis (arterial blood gas pH < 7.35 or HCO_3_ < 15 mmol/L), and 3) elevated plasma lactate level (> 5 mmol/L) [17–19]. Patients with infection (including growth of bacteria in the blood culture or urine culture, white blood cell > 10 cells/high power field in urine analysis, skin infection and pneumonia) were excluded. According to KDIGO 2012 [20], chronic kidney disease (CKD) was defined by kidney structure or function abnormalities more than 3 months and was classified based on eGFR into 5 stages: stage 1 (≥ 90 ml/min/1.73 m^2^), stage 2 (60–89), stage 3 (30–59), stage 4 (15–29), and stage 5 (< 15).

### Statistical analysis

Baseline characteristic of the patients including demographic data, personal history, disease severity, dialysis data and lab tests were presented as mean ± standard deviation (SD) for continuous variables and as frequency (%) for categorical variables. Chi-square test was used to analyze categorical variables. Student’s t-test was used to compare mean between the two groups. Risk factors for 30-day mortality rate amongst patients with MALA were analyzed using univariate and multivariate Cox regression models and presented as the hazard ratios (HR) and the associated 95% confidence interval (CI). Risk factors identified by univariate analysis (p<0.1) were put into the multivariate analysis. The correlations between risk factors were tested using Pearson’s correlation (r). The estimated survival probability of patients between time to dialysis less than 6 and 6 hours or longer, and amongst dialysis modes (PD, HD, and combined dialysis) was analyzed using log-rank test and illustrated by Kaplan-Meier curve. Statistical significance was considered as p<0.05, and all reported probability tests were two-sided. The statistical analysis was conducted using IBM SPSS Statistics for Windows, Version 24.0 (Armonk, NY: IBM Corp).

## Results

Of 105 included patients, mean age was 61.5 ± 11.3 years and 38.1% was male. Most patients (95.2%) were diagnosed acute kidney injury (AKI) stage 3 according to KDIGO 2012 definition [21]. The 30-day mortality rate was 36.2% and the majority (60.5%) of death occurred within 5 days after diagnosis. Ninety patients (85.7%) received dialysis treatment, including all non-survivors.

The demographic characteristics of the patients were presented in **Table 1**. Serum lactate, BUN, creatinine, and potassium levels on admission were comparable between survivors and non-survivors. Proportion of underlying hypertension was higher in non-survivors, on the contrary to underlying CKD. Moreover, disease severity including APACHE II score, rate of ICU admission, haemodynamic instability, and mechanical ventilator requirement was higher in non-survivors. Regarding dialysis, median time to dialysis was 9.05 (IQR: 5.55–16.00) hours. The proportion of patients who received dialysis within 6 hours (early dialysis) and HD treatment was higher in survivors.

**Table 1:** Baseline characteristics of patients with metformin-associated lactic acidosis.

Characteristics	All patients	30-day mortality		*p*-value
		Survivors N = 67 (63.8)	Non-survivors N = 38 (36.2)	
Age (years), mean±SD	61.5±11.3	60.6±11.6	63.1±10.6	0.274
Male, N (%)	40 (38.1)	29 (43.3)	11 (28.9)	0.146
**Underlying diseases**				
Hypertension, N (%)	88 (83.8)	53 (79.1)	36 (94.7)	0.032
Stroke, N (%)	5 (4.8)	3 (4.5)	2 (5.3)	0.856
Cardiovascular diseases, N (%)	4 (3.8)	3 (4.5)	1 (2.6)	0.635
Chronic kidney disease, N (%)	41 (39.0)	32 (47.8)	9 (23.7)	0.015
Stage of chronic kidney disease, N (%) • stage 1 • stage 2 • stage 3 • stage 4 • stage 5	0 (0.0) 5 (4.8) 33 (31.4) 3 (2.9) 0 (0.0)	0 (0.0) 5 (15.6) 24 (75.0) 3 (9.4) 0 (0.0)	0 (0.0) 0 (0.0) 9 (66.7) 0 (0.0) 0 (0.0)	0.248
Creatinine baseline (mg/dL), mean±SD	1.11±0.34	1.14±0.37	1.02±0.23	0.110
eGFR (mL/min/1.73m^2^), mean±SD	65.2±21.5	65.0±23.8	65.8±15.5	0.872
**Current medication**				
Metformin (mg), N (%) • <1000 • 1000–2000 • >2000	4 (3.8) 59 (56.2) 42 (40.0)	2 (3.0) 36 (53.7) 29 (43.3)	2 (5.3) 23 (60.5) 13 (34.2)	0.598
SUs, N (%)	72 (68.6)	47 (70.1)	25 (65.8)	0.644
ACEIs, N (%)	63 (60.0)	39 (58.2)	24 (63.2)	0.619
ARBs, N (%)	8 (7.6)	3 (4.5)	5 (13.2)	0.107
CCBs, N (%)	33 (31.4)	19 (28.4)	14 (36.8)	0.368
NSAIDs, N (%)	6 (5.7)	6 (9.0)	0 (0.0)	0.057
Diuretics, N (%)	27 (25.7)	16 (23.9)	11 (28.9)	0.568
**Initial blood tests**				
BUN (mg/dL), mean±SD	67.23±24.50	71.95±23.30	60.92±22.74	0.021
Creatinine (mg/dL), mean±SD	9.37±3.85	10.43±3.57	7.49±3.66	<0.001
Potassium (mmol/L), mean±SD	6.09±1.20	6.30±1.21	5.72±1.09	0.017
Bicarbonate (mmol/L), mean±SD	5.32±2.96	5.78±2.81	5.22±3.24	0.793
pH, mean± SD	6.95±0.16	6.97±0.16	6.92±0.17	0.107
Lactate (mmol/L), mean±SD	13.22±5.37	12.71±5.00	14.10±5.94	0.204
**Disease severity**				
Stage of acute kidney injury, N (%) • stage 1 • stage 2 • stage 3	2 (1.9) 3 (2.9) 100 (95.2)	0 (0.0) 0 (0.0) 67 (100.0)	2 (5.3) 3 (7.9) 33 (86.8)	0.010
APACHE II score, mean±SD	25.8±6.6	24.4±6.0	28.3±6.9	0.003
ICU admission, N (%)	76 (72.4)	44 (65.7)	32 (84.2)	0.041
Haemodynamic instability, N (%)	63 (60.0)	34 (50.7)	29 (76.3)	0.010
Mechanical ventilator, N (%)	80 (76.2)	46 (68.7)	34 (89.5)	0.016
**Dialysis profiles**				
Dialysis, N (%)	90 (85.7)	52 (77.6)	38 (100.0)	0.002
Indication for dialysis • Volume overload, N (%) • Metabolic acidosis, N (%) • Uremia, N (%) • Hyperkalemia, N (%)	5 (4.8) 89 (84.8) 3 (2.9) 55 (52.4)	2 (3.0) 51 (76.1) 2 (8.0) 34 (50.7)	3 (7.9) 38 (100.0) 1 (5.0) 21 (55.3)	0.256 0.001 0.688 0.656
Time to dialysis (hours), N (%) • <6 hours • ≥6 hours	33 (36.7) 57 (63.3)	25 (48.1) 27 (51.9)	8 (21.1) 30 (78.9)	0.009
Mode of dialysis, N (%) • Peritoneal dialysis • Haemodialysis • Combined	57 (63.3) 24 (26.7) 9 (10.0)	25 (48.1) 21 (40.4) 6 (11.5)	32 (84.2) 3 (7.9) 3 (7.9)	0.001

ACEIs angiotensin-converting enzyme inhibitors, APACHE II Acute Physiology and Chronic Health Evaluation II, ARBs angiotensin II receptor antagonists, BUN blood urea nitrogen, CCBs calcium channel blockers, NSAIDs non-steroidal anti-inflammatory drugs, SU sulfonylureas.

To examine the association between risk factors and disease mortality, high APACHE II score, ICU admission, haemodynamic instability, mechanical ventilator requirement, and time to dialysis 6 hours or longer (late dialysis) and peritoneal dialysis were significantly associated with higher risk of 30-day mortality, using univariate Cox regression model. Chronic kidney disease, high serum BUN, creatinine and potassium levels seemed to be protective factors of disease mortality. Additionally, compared to PD, HD was significantly associated with lower risk of 30-day mortality, using univariate Cox regression model (**Table 2**).

**Table 2:** Association between risk factors and 30-day mortality in patients with metformin-associated lactic acidosis, using univariate Cox-regression analysis.

Risk factors	30-day mortality			
	Univariate		Multivariate	
	Hazard ratio (95% CI)	*p*-value	Hazard ratio (95% CI)	*p*-value
Hypertension	3.82 (0.92–15.87)	0.065	1.95 (0.44–8.51)	0.377
Chronic kidney disease	0.41 (0.19–0.87)	0.020	-	-
BUN (per 1 mg/dL increment)	0.98 (0.97–0.99)	0.033	-	-
Creatinine (per 1 mg/dL increment)	0.84 (0.77–0.92)	<0.001	-	-
Potassium (per 1 mmol/L increment)	0.73 (0.55–0.96)	0.022	-	-
Stage of acute kidney injury (ref. stage 1) • Stage 2 • Stage 3	1.58 (0.26–9.52) 0.31 (0.08–1.32)	0.619 0.113	-	-
APACHE II score (per 1 point decrement)	0.93 (0.89–0.98)	0.004	0.95 (0.91–0.99)	0.038
ICU admission	2.16 (0.90–5.17)	0.084	-	-
Haemodynamic instability	2.73 (1.29–5.78)	0.009	-	-
Mechanical ventilator	2.99 (1.06–8.44)	0.038	-	-
Metabolic acidosis	27.71 (0.80–966.07)	0.067	-	-
Time to dialysis <6 hours	0.39 (0.18–0.85)	0.018	0.31 (0.14–0.69)	0.004
Mode of dialysis (ref. peritoneal dialysis) • Haemodialysis • Combined	0.17 (0.05–0.55) 0.47 (0.14–1.52)	0.003 0.205	0.20 (0.06–0.67) 0.55 (0.17–1.83)	0.010 0.330

APACHE II Acute Physiology and Chronic Health Evaluation II, BUN blood urea nitrogen, ref. reference.

APACHE II score was moderately to strongly correlated with serum potassium, ICU admission, haemodynamic instability, mechanical ventilator requirement, and metabolic acidosis. Time to dialysis was inversely correlated with chronic kidney disease, serum BUN, creatinine, and potassium. Mode of dialysis was moderately correlated with metabolic acidosis.

**Fig 1:**
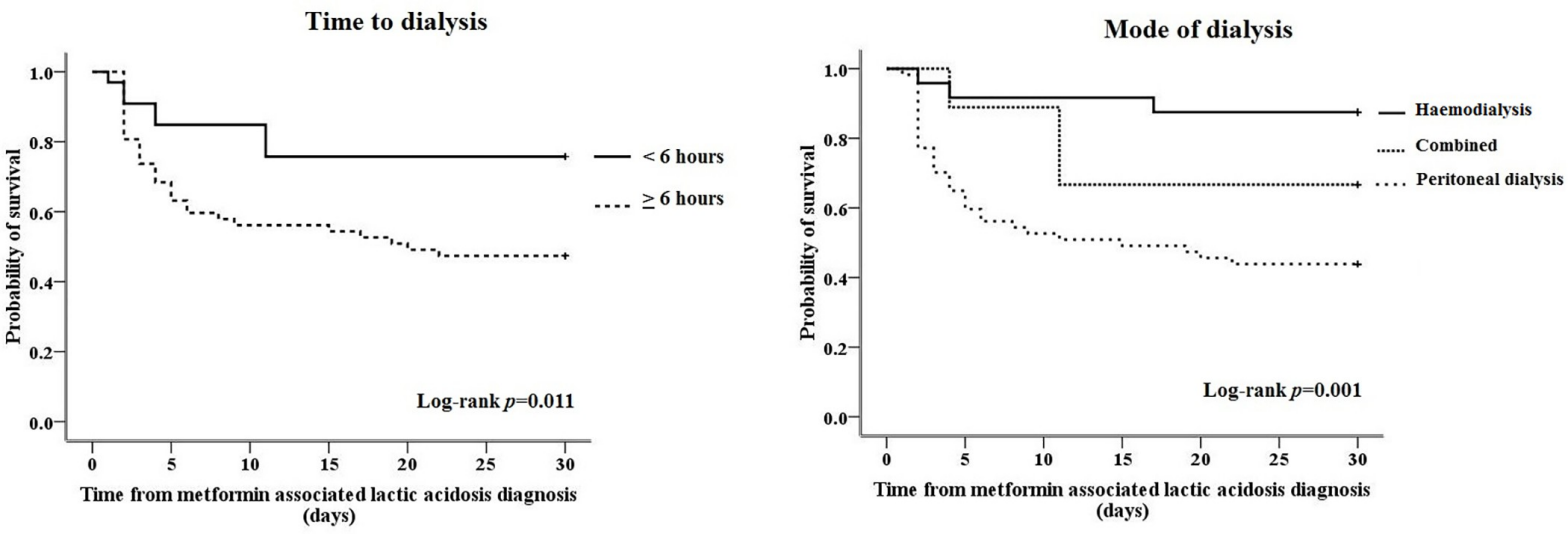
Kaplan-Meier survival function for patients diagnosed with metformin-associated lactic acidosis stratified by a) time to dialysis and b) mode of dialysis.

APACHE II score was moderately to strongly positively correlated with serum potassium (r = 0.41, *p*<0.001), ICU admission (r = 0.37, *p* = 0.005), haemodynamic instability (r = 0.35, *p*<0.001), mechanical ventilator requirement (r = 0.67, *p*<0.001), and metabolic acidosis (r = 0.33, *p* = 0.020). Time to dialysis was inversely correlated with chronic kidney disease (r = -0.22, *p* = 0.041), serum BUN (r = -0.28, *p* = 0.009), serum creatinine (r = -0.36, *p* = 0.001), and serum potassium (r = -0.28, *p* = 0.009). Mode of dialysis was moderately positively correlated with metabolic acidosis (r = 0.33, *p* = 0.018). The correlations between risk factors were presented in **S1 Table**. From multivariate Cox regression analysis, lower APACHE II score (HR = 0.95; 95%CI, 0.91–0.99; p = 0.038), dialysis initiation less than 6 hours (0.31; 0.14–0.69; 0.004) and received HD treatment (0.20; 10.06–0.67; 0.010) were significantly associated with lower 30-day mortality (**Table 2**). Proportion of survivors until 30 days stratified by time to dialysis and dialysis modality amongst patients with MALA was illustrated in **Fig 1**.

## Discussion

This observational study aimed to explore the factors associated with 30-day mortality amongst MALA patients, thus we excluded the patients who had lactic acidosis due to infection. Our study demonstrated hypertension was the most prevalent underlying in patients with MALA and majority of these patients (95.2%) presented with AKI stage 3. The mortality rate of patients with MALA was high and most of them were died within five days. Our study revealed lower APACHE score, early dialysis initiation and HD treatment were independently associated with lower 30-day mortality patients with MALA.

This study showed APACHE II score was associated with higher 30-day mortality. APACHE II score is a validate severity of disease classification system that associated with adverse clinical outcomes in acutely ill patients. Our finding was corresponded to previous studies that revealed APACHE score was associated with an increased risk of death in hospital [15,22]. We also found the correlation between APACHE II score and severity of MALA including increased serum potassium level, metabolic acidosis, haemodynamic instability, mechanical ventilator, and ICU admission. This finding implied that APACHE II score could be a valid prognostic factor for MALA, indifferent to sepsis or other conditions.

The present study showed HD treatment was associated with lower 30-day mortality risk, compared to PD treatment. Previous study of systematic review of case reports and case series [14] showed MALA patients receiving PD tended to have higher mortality rates but not statistically significant; however, the number of patients performed PD was small. Theoretically, HD treatment not only provides higher efficiency to rapidly correct acid-base and electrolyte abnormality but metformin also appears more dialyzable by HD than PD from kinetic data [10]. Despite the fact that we included potential parameters such as haemodynamic instability and severe degree of acidosis or hyperkalemia as confounding factors in the multivariate analysis and found no association with the primary outcome, other factors (e.g., low output heart failure, physician preference, time available, etc.) may remain in decision to select dialysis modality.

Our study showed that MALA patients who received early dialysis within 6 hours after admission were independently associated with lower 30-day mortality risk. Although, recommendation to reduce mortality by early renal KRT in MALA patients has not been well-established, delaying of dialysis may increase exposure time with high plasma metformin and lactate levels leading to poor outcome. Both metformin and lactate are able to be eliminated by KRT [10,23]. Because plasma metformin concentration has been linked to mortality in MALA patients, adverse outcomes could potentially reduce by increasing metformin clearance. Rapid metformin removal would affect both renal recovery and mortality [24]. Metformin, with a molecular weight of less than 500 Da, is widely reported to be dialyzable due to highly water soluble and weakly bound to proteins. However, because of its intracellular penetration, the drug kinetic of metformin was characterized by a large volume of distribution (3.1 liters/kg) [25], which explains why it was difficult to adequately dialyze this culprit drug and achieve efficient and rapid clearance. In fact, dialysis may be most effectiveness if performed before metformin redistributes into the tissues. Additionally, metformin elevates lactate levels via inhibiting mitochondrial respiratory chain complex I and hepatic gluconeogenesis by lowering lactate reuptake [7]. On the contrary to the effect of plasma metformin levels, how serum lactate levels results in mortality was still controversial [24]. Similarly to our study, the recent meta-analysis [26] which resembled in serum lactate level showed lactate was not a good discriminating index between survivors and non-survivors. Besides metformin and lactate removal, dialysis also improved numerous physiologic factors, allowing patients for a rapid recovery [27]. In this study, we determined time to dialysis from admission due to available data. Since presenting symptoms and severity could be different amongst patients when developed MALA, duration between onset of MALA and admission may vary. However, our study supported that early dialysis was the most practical approach for patient’s favorable outcome after adjustment; the lead-time bias as well as misclassification bias may remain occurred.

The association between serum creatinine and mortality in patients with MALA was also still under debated. In this study, serum creatinine, potassium, BUN, together with presence of underlying CKD, were negatively correlated with time to initiate RRT and the association between serum creatinine and mortality risk was not found after conducting multivariable regression analysis. A previous systematic review of case reports for 253 patients [14] showed that higher peak of serum creatinine levels had beneficial effects on survival. As serum creatinine may help clinicians guide to the suspected MALA [14], high serum creatinine levels was associated with favorable outcomes. However, few retrospective studies with smaller sample size did not demonstrate this association [15,24]. Serum creatinine along with the aforementioned factors could be a confounding factor for dialysis initiation on mortality in MALA patients. Hypertension was a factor associated with increased mortality according to the univariate analysis. This probably due to chronic hypertension causes various degree of target organs damage. However, this association was also not demonstrated in the multivariable analysis.

This is the first study showed the associations of dialysis modality and timing of dialysis initiation lower 30-day mortality. This study conducted on the hospital-based data that are systematically and almost completely recorded. MALA diagnosis was confirmed by nephrologists and all patients diagnosed of MALA were counted in this data set. However, this study had some limitations should be concerned. First, due to retrospective study design, some confounding factors and selection bias could affect the study results. Therefore, multivariable analysis was introduced in order to reduce the effect of potential confounding factor and baseline incomparability. Second, the study’s sample size was rather modest; nonetheless, the number of patients in this study is comparable to the previous studies [13,24,28]. Third, this study did not include plasma metformin concentration in the diagnostic criteria of MALA due to unavailability of test and inapplicability in routine care. However, we excluded sepsis and other causes of lactic acidosis. Fourth, the majority of patients in this study were AKI stage 3, thus implement early dialysis initiation before AKI stage 3 should be cautious. Fifth, we included only hospitalized patients who were diagnosed as MALA. Our results would not be generalized to the patients who have a high risk of developing MALA or those who have mild degree of MALA. Last but not least, the application of results must be done judiciously, as this study was restrictively conducted in one major hospital.

In conclusion, mortality rate amongst patients with MALA was high. Early dialysis initiation and HD treatment were associated with lower 30-day mortality. The large scale, well-designed studies are needed to confirm these encouraging results.

## Supporting information

S1 Table Correlation between each pair of risk factors for 30-day mortality in patients with metformin-associated lactic acidosis. (DOCX)

S2 Table Summary data of patients with metformin-associated lactic acidosis. (DOCX)

## Abbreviations

AKI: acute kidney injury
APACHE: acute physiology and chronic health evaluation
BUN: blood urea nitrogen
CI: confidential interval
CKD: chronic kidney disease
HD: haemodialysis
HR: hazard ratio
MALA: metformin-associated lactic acidosis
PD: peritoneal dialysis
KRT: kidney replacement therapies
SD: standard deviation
