# Supplementary material for: Metformin-associated lactic acidosis and factors associated with 30-day mortality
Source: PLoS One. 2022 Aug 30;17(8):e0273678. doi: 10.1371/journal.pone.0273678 (PMC9426915; doi:10.1371/journal.pone.0273678)
Supplement: S1 Table — (DOCX) [file pone.0273678.s001.docx]

**S1 Table. Correlation between each pair of risk factors for 30-day mortality in patients with metformin-associated lactic acidosis**

|  | | **Pearson’s correlation (r)** | ***p*-value** |
| --- | --- | --- | --- |
| Hypertension | Chronic kidney disease | -0.04 | 0.679 |
|  | Blood urea nitrogen | 0.00 | 0.979 |
|  | Creatinine | -0.09 | 0.338 |
|  | Potassium | 0.13 | 0.171 |
|  | APACHE II score | 0.15 | 0.118 |
|  | ICU admission | 0.03 | 0.727 |
|  | Hemodynamic instability | 0.20 | 0.047 |
|  | Mechanical ventilator | 0.01 | 0.904 |
|  | Metabolic acidosis | 0.04 | 0.675 |
|  | Time to dialysis ≥6 hours | 0.02 | 0.886 |
|  | Mode of dialysis | -0.17 | 0.084 |
| Chronic kidney disease | Blood urea nitrogen | 0.26 | 0.007 |
|  | Creatinine | 0.25 | 0.010 |
|  | Potassium | 0.42 | 0.000 |
|  | APACHE II score | 0.16 | 0.096 |
|  | ICU admission | 0.01 | 0.886 |
|  | Hemodynamic instability | 0.02 | 0.872 |
|  | Mechanical ventilator | 0.08 | 0.413 |
|  | Metabolic acidosis | -0.04 | 0.679 |
|  | Time to dialysis ≥6 hours | -0.22 | 0.041 |
|  | Mode of dialysis | 0.17 | 0.078 |
| Blood urea nitrogen | Creatinine | 0.61 | 0.000 |
|  | Potassium | 0.32 | 0.001 |
|  | APACHE II score | 0.16 | 0.110 |
|  | ICU admission | -0.08 | 0.401 |
|  | Hemodynamic instability | 0.06 | 0.559 |
|  | Mechanical ventilator | -0.05 | 0.614 |
|  | Metabolic acidosis | -0.08 | 0.413 |
|  | Time to dialysis ≥6 hours | -0.28 | 0.009 |
|  | Mode of dialysis | -0.05 | 0.636 |
| Creatinine | Potassium | 0.21 | 0.028 |
|  | APACHE II score | -0.08 | 0.431 |
|  | ICU admission | -0.11 | 0.243 |
|  | Hemodynamic instability | 0.00 | 0.998 |
|  | Mechanical ventilator | -0.01 | 0.899 |
|  | Metabolic acidosis | -0.07 | 0.460 |
|  | Time to dialysis ≥6 hours | -0.36 | 0.001 |
|  | Mode of dialysis | 0.07 | 0.449 |
| Potassium | APACHE II score | 0.41 | 0.000 |
|  | ICU admission | 0.01 | 0.953 |
|  | Hemodynamic instability | 0.05 | 0.620 |
|  | Mechanical ventilator | 0.10 | 0.319 |
|  | Metabolic acidosis | -0.03 | 0.770 |
|  | Time to dialysis ≥6 hours | -0.28 | 0.009 |
|  | Mode of dialysis | 0.09 | 0.359 |
| APACHE II score | ICU admission | 0.37 | 0.005 |
|  | Hemodynamic instability | 0.35 | 0.000 |
|  | Mechanical ventilator | 0.67 | 0.000 |
|  | Metabolic acidosis | 0.33 | 0.020 |
|  | Time to dialysis ≥6 hours | -0.17 | 0.117 |
|  | Mode of dialysis | -0.08 | 0.414 |
| ICU admission | Hemodynamic instability | 0.32 | 0.001 |
|  | Mechanical ventilator | 0.56 | 0.000 |
|  | Metabolic acidosis | 0.51 | 0.000 |
|  | Time to dialysis ≥6 hours | -0.07 | 0.496 |
|  | Mode of dialysis | 0.01 | 0.958 |
| Hemodynamic instability | Mechanical ventilator | 0.37 | 0.000 |
|  | Metabolic acidosis | 0.36 | 0.000 |
|  | Time to dialysis ≥6 hours | -0.08 | 0.450 |
|  | Mode of dialysis | -0.08 | 0.445 |
| Mechanical ventilator | Metabolic acidosis | 0.51 | 0.000 |
|  | Time to dialysis ≥6 hours | -0.18 | 0.087 |
|  | Mode of dialysis | -0.06 | 0.577 |
| Metabolic acidosis | Time to dialysis ≥6 hours | -0.08 | 0.450 |
|  | Mode of dialysis | 0.33 | 0.018 |
| Time to dialysis ≥6 hours | Mode of dialysis | -0.04 | 0.687 |
