# Supplementary material for: Metformin-associated lactic acidosis and factors associated with 30-day mortality
Source: PLoS One. 2022 Aug 30;17(8):e0273678. doi: 10.1371/journal.pone.0273678 (PMC9426915; doi:10.1371/journal.pone.0273678)
Supplement: S2 Table — (DOCX) [file pone.0273678.s002.docx]

**S2 Table. Summary data of patients with Metformin-associated lactic acidosis**

| **No.** | **Age (years)** | **Sex** | **HT** | **Mortality** | **Time to death (days)** | **Mode of dialysis** | **Time to RRT (hours)** |
| --- | --- | --- | --- | --- | --- | --- | --- |
| 1 | 82 | female | Yes | Yes | 22 | PD | >6 |
| 2 | 71 | female | Yes | No |  | non-dialysis |  |
| 3 | 52 | female | No | No |  | non-dialysis |  |
| 4 | 15 | male | Yes | No |  | HD | <6 |
| 5 | 53 | male | Yes | No |  | Combined | >6 |
| 6 | 73 | female | Yes | Yes | 4 | PD | >6 |
| 7 | 52 | male | Yes | No |  | PD | <6 |
| 8 | 58 | female | Yes | No |  | non-dialysis |  |
| 9 | 61 | female | No | Yes | 4 | HD | >6 |
| 10 | 51 | female | Yes | No |  | HD | >6 |
| 11 | 71 | female | Yes | No |  | PD | <6 |
| 12 | 54 | male | No | No |  | non-dialysis |  |
| 13 | 71 | male | Yes | No |  | HD | >6 |
| 14 | 40 | male | Yes | Yes | 11 | Combined | <6 |
| 15 | 53 | male | No | No |  | non-dialysis |  |
| 16 | 58 | female | Yes | No |  | non-dialysis |  |
| 17 | 64 | female | Yes | No |  | HD | >6 |
| 18 | 72 | female | No | No |  | HD | >6 |
| 19 | 54 | female | Yes | No |  | PD | <6 |
| 20 | 54 | male | No | No |  | PD | >6 |
| 21 | 60 | female | Yes | No |  | non-dialysis |  |
| 22 | 55 | male | No | No |  | HD | >6 |
| 23 | 68 | male | Yes | No |  | non-dialysis |  |
| 24 | 71 | female | Yes | Yes | 3 | PD | >6 |
| 25 | 78 | male | Yes | No |  | PD | >6 |
| 26 | 54 | female | Yes | No |  | non-dialysis |  |
| 27 | 67 | female | No | No |  | HD | <6 |
| 28 | 52 | male | Yes | Yes | 2 | PD | >6 |
| 29 | 74 | male | Yes | Yes | 20 | PD | >6 |
| 30 | 66 | male | Yes | No |  | non-dialysis |  |
| 31 | 62 | male | Yes | No |  | non-dialysis |  |
| 32 | 69 | female | Yes | No |  | non-dialysis |  |
| 33 | 50 | female | No | No |  | HD | >6 |
| 34 | 62 | female | Yes | No |  | non-dialysis |  |
| 35 | 75 | female | Yes | Yes | 3 | PD | >6 |
| 36 | 61 | female | Yes | No |  | non-dialysis |  |
| 37 | 68 | female | No | No |  | HD | >6 |
| 38 | 75 | female | Yes | Yes | 2 | PD | >6 |
| 39 | 64 | female | Yes | Yes | 4 | Combined | <6 |
| 40 | 64 | male | Yes | No |  | PD | >6 |
| 41 | 60 | female | Yes | Yes | 2 | PD | >6 |
| 42 | 48 | male | Yes | No |  | HD | >6 |
| 43 | 60 | male | Yes | Yes | 5 | PD | >6 |
| 44 | 61 | female | Yes | Yes | 5 | PD | >6 |
| 45 | 70 | female | Yes | No |  | Combined | <6 |
| 46 | 48 | female | Yes | No |  | HD | <6 |
| 47 | 64 | female | Yes | No |  | HD | <6 |
| 48 | 69 | male | Yes | No |  | HD | >6 |
| 49 | 85 | male | Yes | No |  | PD | <6 |
| 50 | 89 | female | Yes | No |  | PD | <6 |
| 51 | 61 | female | No | No |  | PD | <6 |
| 52 | 71 | female | Yes | Yes | 6 | PD | >6 |
| 53 | 54 | female | Yes | No |  | PD | >6 |
| 54 | 53 | female | Yes | No |  | HD | >6 |
| 55 | 74 | female | Yes | Yes | 2 | HD | >6 |
| 56 | 62 | female | Yes | Yes | 6 | PD | >6 |
| 57 | 45 | male | No | No |  | HD | <6 |
| 58 | 76 | female | Yes | Yes | 3 | PD | >6 |
| 59 | 72 | female | Yes | Yes | 2 | PD | >6 |
| 60 | 56 | male | Yes | No |  | HD | >6 |
| 61 | 85 | female | Yes | No |  | PD | <6 |
| 62 | 77 | male | Yes | Yes | 5 | PD | >6 |
| 63 | 60 | female | Yes | Yes | 2 | PD | <6 |
| 64 | 69 | male | Yes | Yes | 11 | PD | <6 |
| 65 | 52 | male | Yes | Yes | 1 | PD | <6 |
| 66 | 63 | female | Yes | Yes | 4 | PD | >6 |
| 67 | 61 | female | Yes | Yes | 2 | PD | >6 |
| 68 | 47 | female | Yes | Yes | 2 | PD | >6 |
| 69 | 68 | female | Yes | Yes | 2 | PD | >6 |
| 70 | 66 | male | Yes | Yes | 9 | PD | >6 |
| 71 | 59 | female | Yes | No |  | PD | <6 |
| 72 | 51 | female | Yes | No |  | PD | <6 |
| 73 | 50 | male | Yes | No |  | PD | >6 |
| 74 | 42 | male | Yes | No |  | PD | >6 |
| 75 | 60 | female | Yes | Yes | 2 | PD | >6 |
| 76 | 56 | male | Yes | No |  | PD | >6 |
| 77 | 57 | male | No | No |  | PD | <6 |
| 78 | 64 | male | Yes | No |  | PD | <6 |
| 79 | 39 | male | Yes | Yes | 11 | Combined | <6 |
| 80 | 48 | female | Yes | No |  | Combined | <6 |
| 81 | 64 | male | Yes | No |  | Combined | <6 |
| 82 | 63 | female | Yes | No |  | Combined | >6 |
| 83 | 58 | female | Yes | No |  | HD | >6 |
| 84 | 66 | male | Yes | No |  | HD | >6 |
| 85 | 59 | female | Yes | Yes | 17 | HD | >6 |
| 86 | 63 | male | Yes | No |  | HD | <6 |
| 87 | 60 | male | Yes | No |  | HD | <6 |
| 88 | 73 | female | No | No |  | PD | <6 |
| 89 | 63 | female | Yes | Yes | 3 | PD | >6 |
| 90 | 70 | female | Yes | No |  | PD | >6 |
| 91 | 64 | female | Yes | No |  | PD | <6 |
| 92 | 86 | male | No | No |  | HD | >6 |
| 93 | 74 | female | Yes | No |  | PD | <6 |
| 94 | 67 | male | Yes | Yes | 15 | PD | >6 |
| 95 | 71 | female | Yes | Yes | 2 | PD | >6 |
| 96 | 56 | female | Yes | No |  | non-dialysis |  |
| 97 | 64 | female | Yes | No |  | Combined | >6 |
| 98 | 54 | female | Yes | Yes | 4 | PD | <6 |
| 99 | 56 | female | Yes | Yes | 19 | PD | >6 |
| 100 | 38 | male | No | Yes | 8 | PD | >6 |
| 101 | 57 | female | Yes | No |  | PD | <6 |
| 102 | 60 | female | Yes | No |  | PD | >6 |
| 103 | 42 | male | Yes | No |  | PD | >6 |
| 104 | 60 | female | Yes | Yes | 2 | PD | <6 |
| 105 | 66 | female | Yes | Yes | 2 | PD | >6 |

HD hemodialysis, HT hypertension, PD peritoneal dialysis, RRT renal replacement therapy
